# Collision of adenocarcinoma and gastrointestinal stromal tumour (GIST) in the stomach: report of a case

**DOI:** 10.1186/1477-7800-4-2

**Published:** 2007-01-12

**Authors:** Iraklis E Katsoulis, Manuela Bossi, Paul I Richman, Jeremy I Livingstone

**Affiliations:** 1 Upper Gastrointestinal Surgery Unit, Watford General Hospital, Watford, UK; 2 Pathology Department, Mount Vernon Cancer Centre, London, UK

## Abstract

A 78-year-old woman was diagnosed with a proximal gastric adenocarcinoma and underwent an elective D2 total gastrectomy with splenectomy. Subsequent histopathology revealed the presence of another tumour at the gastric antrum. This was a small benign gastrointestinal stromal tumour (GIST) mixed with gastric adenocarcinoma cells similar to those of the main gastric tumour i.e. a collision tumour. The literature has only few previous reports of this very rare association. It is not known whether this synchronicity is incidental or there is a causative factor inducing the development of tumours of different histotypes in the same organ. Pathologists, oncologists and surgeons should be aware of this interesting condition.

## Background

Adenocarcinoma is the most common histological type of gastric tumour. It may coexist with another synchronous tumour of different histological type in a different part of the stomach. Gastric adenocarcinoma may coexist most commonly with lymphoma and less commonly with carcinoid and gastrointestinal stromal tumour (GIST). Rarely, cells of different histological types may intermix and form a collision tumour in the stomach. We present here the very rare combination of a synchronous proximal gastric adenocarcinoma and a distal gastric collision tumour consisting of GIST and adenocarcinoma cells similar to those of the main gastric tumour.

## Case Presentation

A 78-year-old woman presented with a 6-month history of dyspeptic symptoms, epigastric pain and weight loss. Gastroscopy showed mucosal nodularity and ulceration at the proximal gastric body with an "hour glass" deformity, an appearance suggestive of malignancy. Multiple mucosal biopsies were obtained and histopathology revealed a poorly differentiated adenocarcinoma and chronic gastritis. Computed tomography of the abdomen showed diffuse thickening of the gastric wall and a few enlarged lymph nodes in the lesser sac.

The patient underwent an elective D2 total gastrectomy with splenectomy as the bulky gastric tumour was extending into the splenic hilus and a Roux-en-Y reconstruction was performed.

**Figure 1:**
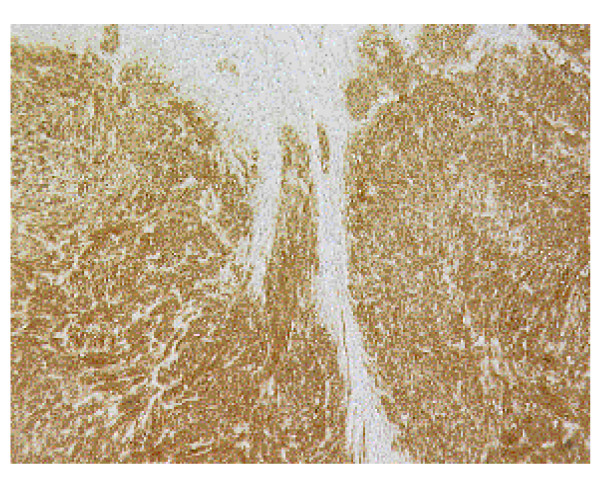
Gastrointestinal stromal tumour showing CD117 expression. Immunohistochemistry.

**Figure 2:**
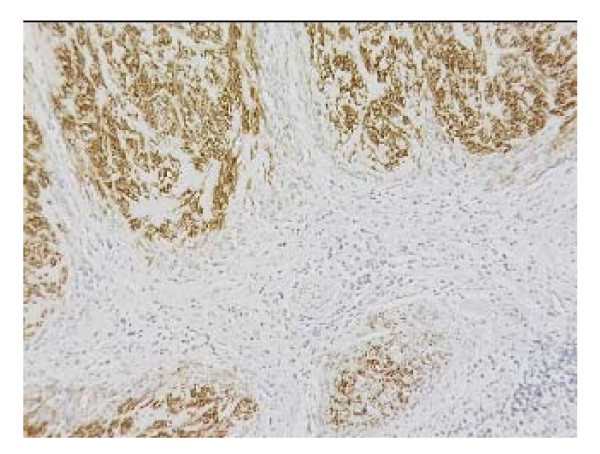
Gastrointestinal stromal tumour showing desmin expression. Immunohistochemistry.

**Figure 3:**
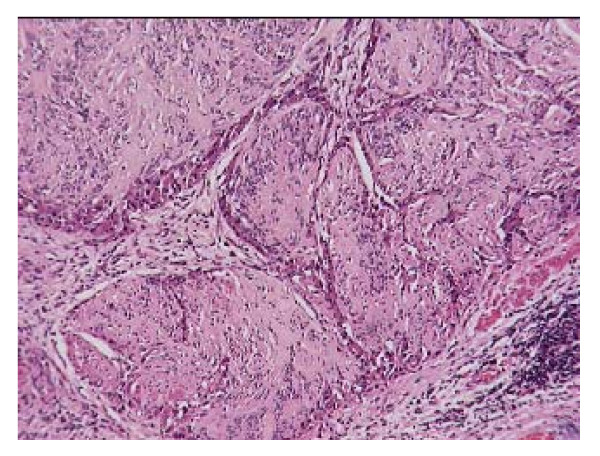
Nodules of gastrointestinal stromal tumour (GIST) composed of spindle cells. There is infiltration around the periphery of the nodules by carcinoma cells (staining slightly more deeply pink). Haematoxylin and Eosin.

**Figure 4:**
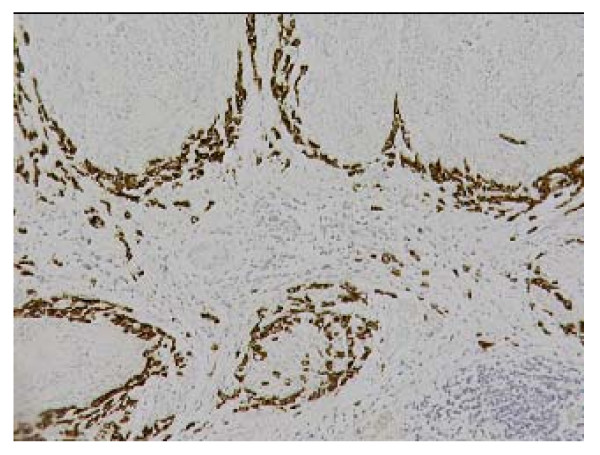
Nodules of gastrointestinal stromal tumour showing peripheral infiltration by carcinoma cells. Cytokeratin immunohistochemistry.

The histopathological examination of the specimen macroscopically showed firm texture of the proximal stomach and vague nodular appearance of the mucosa over a 100 by 80 mm area. Microscopically the proximal stomach showed transmural infiltration by poorly differentiated diffuse adenocarcinoma, which had reached the serosal surface. There was vascular invasion, infiltration of 10 out of 34 nodes and numerous extranodal tumour deposits. A nodule of firm white tissue, 9mm diameter, was present on the external surface of the gastric antrum. Histological examination revealed this nodule to be a benign gastrointestinal stromal tumour (GIST), which had arisen from the muscularis propria. It was composed of interwoven cytologically bland spindle shaped cells that were demonstrated by immunohistochemistry to be uniformly positive for CD117. Scattered spindle cells of the GIST also expressed smooth muscle actin, desmin and S-100 (Fig. 1, 2, 3). Cytoceratin immunohistochemistry was negative. An additional finding was the presence within the GIST of numerous cytokeratin positive polygonal-shaped gastric carcinoma cells (Fig. 4). These cells were cytologically similar to those of the main gastric tumour. They were seen mainly around the peripheral parts of the GIST but were also present within its centre.

## Discussion

There are only a few previous reports of simultaneous adenocarcinoma and GIST in the stomach [1-3]. In these cases the synchronous tumours were located in different parts of the stomach. In our case there was a proximal gastric adenocarcinoma and a distal gastric GIST. Interestingly, however, gastric adenocarcinoma cells similar to those of the main tumour were also found within the GIST. They were seen mainly around the peripheral parts of the GIST but were also present within its centre.

GISTs are usually sessile, big, soft tumours and can develop necrosis or ulceration of the overlying mucosa. However, when the GIST is submucosal or subserosal the gastric mucosa may not be invaded and the endoscopic biopsies can be normal. In most of the reported cases of synchronous gastric adenocarcinoma and GIST, the preoperative biopsy fragments showed only adenocarcinoma and the GIST were detected only following laparotomy and examination of the resected stomachs. In our case the total gastrectomy was performed for the proximal gastric adenocarcinoma and a small GIST was found incidentally with the histopathological examination of the specimen. The coexistence of primary gastric adenocarcinoma and GIST has often been detected incidentally on gastric mucosa or serosa, or occasionally intramurally, at surgery or gastroscopy for other reasons.

Gastric collision tumours are uncommon and most are composed of adenocarcinoma intermixed with gastric lymphoma [4-6] or with carcinoid tumours [7]. The collision of adenocarcinoma and GIST in the stomach is extremely rare and to the best of our knowledge only two cases have been reported previous to the present report [8,9].

Various hypotheses have been proposed regarding the simultaneous development of GIST and adenocarcinoma. It is not known whether or not this association is a simple incidental coexistence or whether the two lesions are connected by a causal relationship. It has been suggested that gene mutations or a single carcinogenic agent might interact with two neighbouring tissues resulting in the development of tumours of different histotypes in the same organ [1]. In the particular case of a collision gastric tumour, it has been hypothesized that the same carcinogen may induce simultaneous proliferation of different cell lines i.e. epithelial and stromal cells.

## Competing interests

The authors declare that they have no competing interests.

## Authors' contributions

IEK conceived the idea, contributed to the literature search and wrote

the final manuscript.

MB performed the literature search and wrote the case presentation.

PIR contributed to the writing of the case presentation and provided the figures.

JIL revised the manuscript.

All authors read and approved the final manuscript.
